# A targeted siRNA‐loaded PDL1‐exosome and functional evaluation against lung cancer

**DOI:** 10.1111/1759-7714.14445

**Published:** 2022-05-11

**Authors:** Xianbin Lin, Liangan Lin, Jingyang Wu, Wentan Jiang, Jiayun Wu, Jianshen Yang, Chun Chen

**Affiliations:** ^1^ Department of Thoracic Surgery The Second Affiliated Hospital of Fujian Medical University Quanzhou China; ^2^ Department of Thoracic Surgery, Fujian Medical University Union Hospital Key Laboratory of Cardio‐Thoracic Surgery (Fujian Medical University) Fuzhou China

**Keywords:** exosome, lung cancer, nanocarrier, PD‐L1, siRNA

## Abstract

**Background:**

As an endocytic nanosicle involved in intercellular communication, an exosome can efficiently deliver drugs from one cell to another and deliver therapeutic short interfering RNA (siRNA) to target cells. This is conducive to gene therapy for cancers. In this study, an exosome was used as the siRNA‐loaded substrate to prepare a targeted siRNA‐loaded PD‐L1 exosome and evaluate its function against lung cancer.

**Methods:**

The optimal preparation process and binding ratio of the targeted nanovesicle/siRNA complex was determined by detecting the particle size, potential, and other physical parameters in combination with cell binding and uptake capacity of exosome complexes. The biological cell behavior of targeted exosome nanosicles was evaluated through cytotoxicity, apoptosis, and the cell uptake capacity.

**Results:**

A targeted exosome nanovesicle capable of loading siRNA and characterized with low toxicity, high loading rate, and the ability to be used for targeted tumor cell gene therapy was constructed.

**Conclusion:**

The PD‐L1 targeting exosome can be used as an efficient siRNA delivery carrier, which is an efficient and safe nanocarrier for tumor targeted gene therapy.

## Graphical abstract

A targeted exosome nanovesicle capable of loading siRNA and characterized with low toxicity, high loading rate and the ability of targeted gene therapy for tumor cells was constructed to effectively inhibit lung cancer cells.

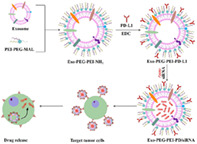

## INTRODUCTION

Lung cancer is the second most common malignant tumor in the world, with ~1.8 million deaths in 2020, making it the greatest threat to human health.^1^ Although the development of lung cancer is associated with multiple genetic mutations and heterogeneity, surgical removal is still the most effective therapy. Surgical intervention is ineffective for advanced lung cancer treatment. Targeted immunotherapy and gene therapy are effective therapies for lung cancer.^2^, ^3^ Immunotherapy is rapidly developing into a promising anticancer treatment strategy. Conventional cancer drugs usually target cancer cells directly, while immunotherapy activates immune cells to identify and eradicate tumor cells. Since PD‐1/PD‐L1 is one of the most critical immune checkpoints, blocking the PD‐1/PD‐L1 pathway has become the primary focus for cancer immunotherapy. PD‐L1 is expressed on the surface of tumor cells and can bind to PD1 molecules on the surface of tumor‐infiltrating lymphocytes, inhibiting the function of lymphocytes, and ultimately leading to tumor immune escape.^4^ Using immunohistochemistry (IHC), cell lines and animal experiments combined with clinical data and other methods show that PD‐L1 is highly expressed in many tumor tissues. These include a variety of high incidence solid tumors, such as lung cancer, liver cancer, stomach cancer, and colorectal cancer. In addition, it is associated with the poor prognosis of these tumors.^5^, ^6^, ^7^, ^8^. Studies showed that PD‐L1 is highly expressed in the cell membrane and cytoplasm of cancer cells, while it demonstrates low level to zero expression in paracancerous tissues and normal tissues. Therefore, PD‐1/PD‐L1 can be used as a very effective target for targeted delivery of drugs and can be used for the modification of carrier materials.^9^, ^10^, ^11^

In recent years, the development of RNA‐based therapies has been greatly enhanced. Specifically triggering RNA interference (RNAi) has been one of the most widely used techniques in biomedical applications. RNAi takes advantage of posttranscriptional sequence‐specific gene silencing to process double‐stranded RNA into small interfering RNA (siRNA) and selectively cut target mRNA as part of the RNA‐induced silencing complex. After the finding that synthetic siRNA can be exogenously introduced into cells to activate RNAi, this method has become an effective method to inhibit specific target genes in different domains. Gene silencing induced by siRNA opened up a new pathway for drug development.^12^, ^13^ Exosomes are small vesicles ~30–150 nm in diameter, secreted by living cells, and demonstrate a typical lipid bilayer structure. They are involved in the communication between cells, transport of substances, the maintenance of normal physiological processes, and are associated with the development of diseases.^14^, ^15^ Studies showed that the exosome is an endocytic nanosicle involved in cell–cell communication. It can efficiently deliver drugs between cells and deliver therapeutic siRNA to target cells to facilitate cancer gene therapy.^16^, ^17^, ^18^ However, challenges still exist for the clinical application of exosomes.

In this study, attempts were made to use exosome as the basic carrier material to modify PEG‐PEI on the surface of the exosome to result in exosome‐PEG‐PEI (Exo‐PEG‐PEI). This would provide it with a long circulation time and positive electricity, thus making it capable of carrying siRNA for gene delivery. Meanwhile, the PD‐L1 antibody was modified on the surface of the exosome to construct exosome‐PEG‐PEI‐PD‐L1 (Exo‐PEG‐PEI‐PD), allowing the exosome to exhibit higher tumor targeting and identification capabilities. In this study, a series of in vitro evaluations of cytotoxicity and tumor cell recognition and inhibition was carried out with the Exo‐PEG‐PEI‐PD/siRNA complex using cell experiments. Also, the functions of the compound drug constructed were evaluated experimentally. The results indicate that the PD‐L1 targeting exosome can be used as an efficient siRNA delivery carrier, which is an efficient and safe nanocarrier for tumor targeted gene therapy.

## METHODS

### Cells and materials

Cell lines (A549 and H460) of human nonsmall cell lung cancer (NSCLC) and human umbilical vein endothelial cells (HUVEC) were purchased from Shanghai GenePharma Co., Ltd. DMEM culture solution, fetal bovine serum, and trypsin were purchased from Gibco. PEG‐PEI‐MAL, 1‐ethyl‐3‐(3‐dimethylaminopropyl) carbodiimide (EDC) were purchased from Huzhou Liyuan Medical Laboratory Co., Ltd. Next, the PD‐L1 antibody was purchased from Shanghai GenePharma Co., Ltd. GAPDH was purchased from Cell Signaling Technologies. The OLYMPUS B × 61 fluorescence microscope was purchased from Olympus Corporation. The flow cytometer BD FACS caliber was purchased from BD. The TALOS F200X electron microscope was purchased from Thermo Fisher Scientific. The Zetasizer Nano ZS was purchased from Malvern Panalytical. Finally, the ND‐1000 NanoDrop was provided by Caliber Technology Development Co., Ltd.

### Separation, purification, and characterization of extracellular vesicles

A549 cells were cultured for 2 days, and the cells and cell culture solution were then collected. Exosomes were collected by differential centrifugation at 15 000 *g* for 3 min to remove cells, cell debris, and microvesicles. In order to precipitate the exosomes, they were then subjected to ultracentrifugation at 100 000 *g* for 70 min,^19^, ^20^ which were collected in phosphate‐buffered saline (PBS) and stored at −80°C. Then, 20 μl of exosome diluent was removed and observed using transmission electron microscopy (TEM). One milliliter of exosomes was removed to test the particle size and the potential distribution. The presence of tetraspanins on the outer surface of the exosomes was determined by a plate‐immobilization of purified exosomes.^21^

### Material preparation and characterization

Using the previously described methods, the exosome‐PEG‐PEI‐MAL complex (Exo‐PEG‐PEI) was prepared.^22^ PEG‐PEI‐maleimide (PEG‐PEI‐MAL) (10 μl at 300 μg/ml) was added to a 60 μl exosome aliquot, and made up to 50 μl with PBS. It was incubated without agitation in the dark at room temperature for 60 min, after which the PD‐L1 antibody was added, and the antibody was coupled with Exo‐PEG‐PEI using the EDC coupling agent to form the (Exo‐PEG‐PEI‐PD‐L1). siRNA transfected with a green fluorescent label (the sequence of PD‐L1 siRNA (sense) 5′‐CCAGCACACUGAGAAUCAATT‐3′ and (antisense) 5′‐UUGAUUCUCAGUGUGCUGGTT‐3′) was incubated with Exo‐PEG‐PEI‐PD‐L1 at room temperature for 30 min.^23^ This resulted in the targeted siRNA‐loaded exosome (Exo‐PEG‐PEI‐PD/siRNA). The particle size and potential of the materials were measured using a Malvern laser particle analyzer (ZetasizerNanoZS). All measurements were performed three times and averaged. The organizational structure and surface morphology of the materials were observed using transmission electron microscopy (TEM). Also, an infrared spectrometer was used to test the materials.

### siRNA release and phagocytosis assay

A series of siRNA standard solutions with a concentration gradient was prepared. The maximum absorption wavelength of siRNA was determined by scanning the full spectrum with an ultraviolet spectrophotometer. The regression equations were obtained from the standard curves based on concentration (C) and absorbance (ABS). Afterwards, 1 mg of the material complex was removed and dispersed in 10 centrifuge tubes filled with 1 ml PBS solution, to which 20, 40, 60, 80, and 100 μg of siRNA was added, respectively. When the solution was fully dispersed, it was placed in a thermostatic oscillator for oscillation‐recombination at 70 rpm for 6 h. Then, it was centrifuged to remove the supernatant to test its absorbance and calculate the load according to the regression equation.

The material above with the best experimental effect was used for the release experiment. A total of 3 mg of material was weighed and dispersed in 3 ml PBS buffer solution with a pH value of 6 and 7. The sample was placed in a thermostatic oscillator set at 37°C, 70 rpm. Each group was provided with three duplicate samples and time points every 2 h for 24 h and then daily up to 7 days. The samples were centrifuged, and 3 ml of supernatant was used to measure the absorbance and calculate the release rate of siRNA.

Healthy A549 and H460 cells were added to a 24‐well microplate at 1 × 10^5^ cells/well with 1 ml Dulbecco's modified eagle medium (DMEM) high sugar medium for 24 h. After the cells attached to the well, the medium was discarded, and different materials were added containing siRNA labeled with a carboxyl fluorescein reagent. After 12 h of culture, 20 μl DAPI was added. DiIC16 (Sigma‐Aldrich) was used to label the exosome, and immunofluorescence microscopy was used to compare the endocytosis efficiency of siRNA with different material loads.

### Effects of cytotoxicity and proliferation

Adherent HUVECs and A549 were cultured with PBS, and different concentrations of materials were added for coculture and incubated for 24 h. Then, MTT was added to each well and removed after 4 h incubation. Next, 800 μl dimethyl sulfoxide (DMSO) was added to each well to fully dissolve the formazan, and 200 μl of the solution was transferred into 96‐well microplates. The absorbance value was measured using an enzyme‐linked immunosorbent assay (ELISA) microplate reader, and the cell survival rate was calculated. A549 and H460 cells were cultured until they were adherent, then the medium was removed, and the materials with a concentration of 10 μg/ml prepared with the medium were added. After different incubations times with the mixture, 20 μl MTT (5 μg/ml) was added to each well and then removed after 4 h of incubation. Then, 200 μl DMSO was added to each well to fully dissolve the formazan. The absorbance value was measured using an ELISA microplate reader, and the cell proliferation rate was calculated. A549 and H460 cells were cultured as above, and the material (10 μg/ml) was added. After 24 h of culture, the culture medium was replaced with a medium containing 10 μmol/l EdU and incubated for 1 h, fixed and permeablized, Apollo stain was added, and then DAPI staining was added to visualize the nucleus. Finally, the collected images were observed with a fluorescence microscope to detect the rate of cell proliferation.

### Effects of apoptosis and cloning

A549 and H460 cells were cultured to be adherent to the logarithmic phase, and then, the medium was removed. The materials (10 μg/ml) were added, and the cells were incubated for 24 h. Then, according to the manual for the apoptosis kit, annexin V‐FITC, and propidium iodide staining solution were added. The blended mixture was incubated in the dark at room temperature for 20 min. The apoptosis rate was determined by flow cytometry. Adherent logarithmic phase A549 and H460 cells were incubated with the prepared material (10 μg/ml). After 24 h incubation, trypsin was used to digest the cells into single cells. They were put into a dish containing 10 ml of prewarmed culture medium at 37°C and incubated for 2 weeks. When macroscopically visible clones were found in the petri dish, cells were washed with PBS and fixed. They were dyed with crystal violet, rinsed with distilled water, and dried. Photographs were used to calculate the clone formation rate.

### Detection of the PDL1 expression level

A549 and H460 cells were inoculated into 6‐well plates to a density of 2 × 10^5^ cells per well, and siRNA, Exo‐PEG‐PEI‐siRNA, and Exo‐PEG‐PEI‐PD/siRNA were added into each group, respectively. The siRNA concentration was 100 nmol, and cells in each group were collected after 2 days. For qPCR detection, samples were prepared according to the qPCR mixture reagent specifications. GAPDH was the internal reference gene, and primer sequences were as follows: PDL1: forward primer, 5′‐CAAGTAGACAAGCTTCCACCATGAGGATATTTGCT‐3′; reverse primer, 5′‐CGTTCGTCGACTGCGTCTCCTCGAATTG‐3′; GAPDH: forward primer, 5′‐GTCAACGGATTTGGTCTGTATT‐3′; reverse primer, 5′‐AGTCTTCTGGGTGGCAGTGAT‐3′. For qPCR samples, 10 μl 2 × mixture, 0.3 μl upstream and downstream primers (10 μmol/l), and 1.5 μl template cDNA, to a total volume of 20 μl, with the addition of 7.9 μl RNase‐free water were added to tubes. After evenly mixing and centrifugation, qPCR was done using a Bio‐Rad qPCR instrument. The run cycle was as follows: predenaturation at 94°C for 10 min, the amplification at 94°C for 30 s, 55°C for 30 s and 72°C for 30 s, for a total of 40 cycles. GAPDH was used as the internal reference gene, and the results were analyzed by the relative quantitative 2^−ΔΔCt^ method. For western blot detection, radioimmunoprecipitation assay buffer (RIPA) was used to lyse the cells and the extract proteins. Protein samples were concentrated and separated by electrophoresis on 12% SDS PAGE gels and transferred to polyvinylidene difluoride (PVDF) membranes. After antibody incubation, western blots were imprinted on a PVDF membrane using chemiluminescence for detection.

### Statistical analysis

The statistical software SPSS 21.0 was used for analysis. A *t*‐test was used for comparison between the two groups. The differences were statistically significant if *p* < 0.05.

## RESULTS

### Preparation flow

Polyethyleneimine (PEI) exhibits a large number of free primary amine groups in its molecular structure. These primary colloidal groups can be protonized to make the surface of PEI molecules charged with high density positive charges. PEI can electrochemically combine with polyethylene glycol (PEG) and with negatively charged nucleic acid molecules (including DNA and RNA) to efficiently transfect cells. Therefore, PEI is an efficient siRNA nonviral delivery vector.^24^, ^25^ In this study, Exo‐PEG‐PEI‐PD/siRNA was prepared in three steps. In step 1, to obtain the aminated Exo‐PEG‐PEI, the exosome reacted with PEG‐PEI‐MAL under certain conditions. In step 2, the carboxylated PD‐L1 antibody and coupling agent EDC were added to form Exo‐PEG‐PEI‐PD‐L1. Finally, in step 3, siRNA was loaded to obtain Exo‐PEG‐PEI‐PD/siRNA.

### Identification and material characterization of exosomes

**FIGURE 1:**
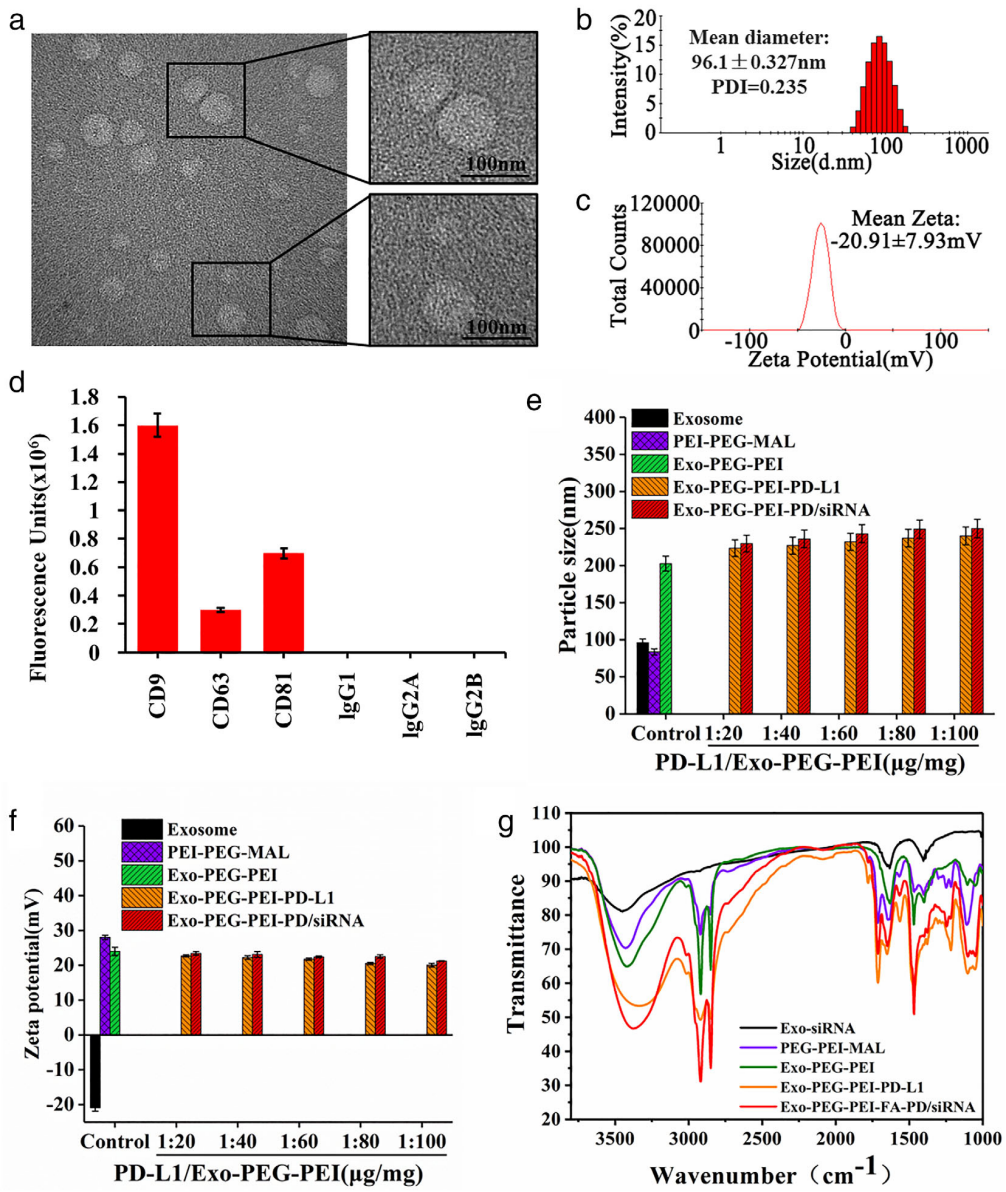
Identification and material characterization of exosomes. (a) Transmission electron microscopy (TEM) test for the exosomes. (b) Particle size distribution of exosomes. (c) Potential distribution of exosomes. (d) Plate‐immobilized exosomes with surface staining for tetraspanin proteins. (e) Particle size distribution of materials. (f) Potential distribution of materials. (g) Infrared test on materials

Under TEM, exosomes were regular balls 30–150 nm in size (Figure 1a), with an average particle size of 96.1 nm (Figure 1b) and a charge of −20.91 mV (Figure 1c). Exosomes fixed on microdroplet plates were stained with antibodies against CD9, CD63, CD81, and the corresponding isotype controls. The results showed that strong and specific signals occurred from exosomal‐specific markers (Figure 1d). All of these are special characteristics of exosomes.^22^ The particle size of Exo‐PEG‐PEI was 205 nm. The range of the particle size of complexes with different PD‐L1/Exo‐PEG‐PEI proportions in Exo‐PEG‐PEI‐PD‐L1 and Exo‐PEG‐PEI‐PD/siRNA was 230–250 nm (Figure 1e), indicating that the complexed particles are small and relatively stable in size. The complexes with different PD‐L1/Exo‐PEG‐PEI proportions in Exo‐PEG‐PEI, Exo‐PEG‐PEI‐PD‐L1, and Exo‐PEG‐PEI‐PD/siRNA were positively charged and ranged from 20 to 25 mV (Figure 1f). At present, siRNA is mainly delivered by cationic carrier materials, and the nanocomposites were formed by siRNA. Cationic carriers can load siRNA drugs through positive and negative electric interactions, to help the delivery system to escape lysosomal degradation.^26^, ^27^ The results of infrared testing showed that PEG‐PEI‐MAL, Exo‐PEG‐PEI, Exo‐PEG‐PEI‐PD‐L1, and Exo‐PEG‐PEI‐PD/siRNA demonstrated a distinct absorption peak at the ‐C‐N‐ characteristic peak of 1425.69, the ‐C‐H characteristic peak of 2917.67, and the ‐C=O‐H characteristic peak of 2846.74, indicating that all of them demonstrated the characteristics of the PEG‐PEI complex. Exo‐PEG‐PEI‐PD‐L1 and Exo‐PEG‐PEI‐PD/siRNA demonstrated the highest similarity and were different from PEG‐PEI‐MAL and Exo‐PEG‐PEI, indicating that the PD‐L1 antibody was successfully coupled (Figure 1g).

### SiRNA loading and release assay

**FIGURE 2:**
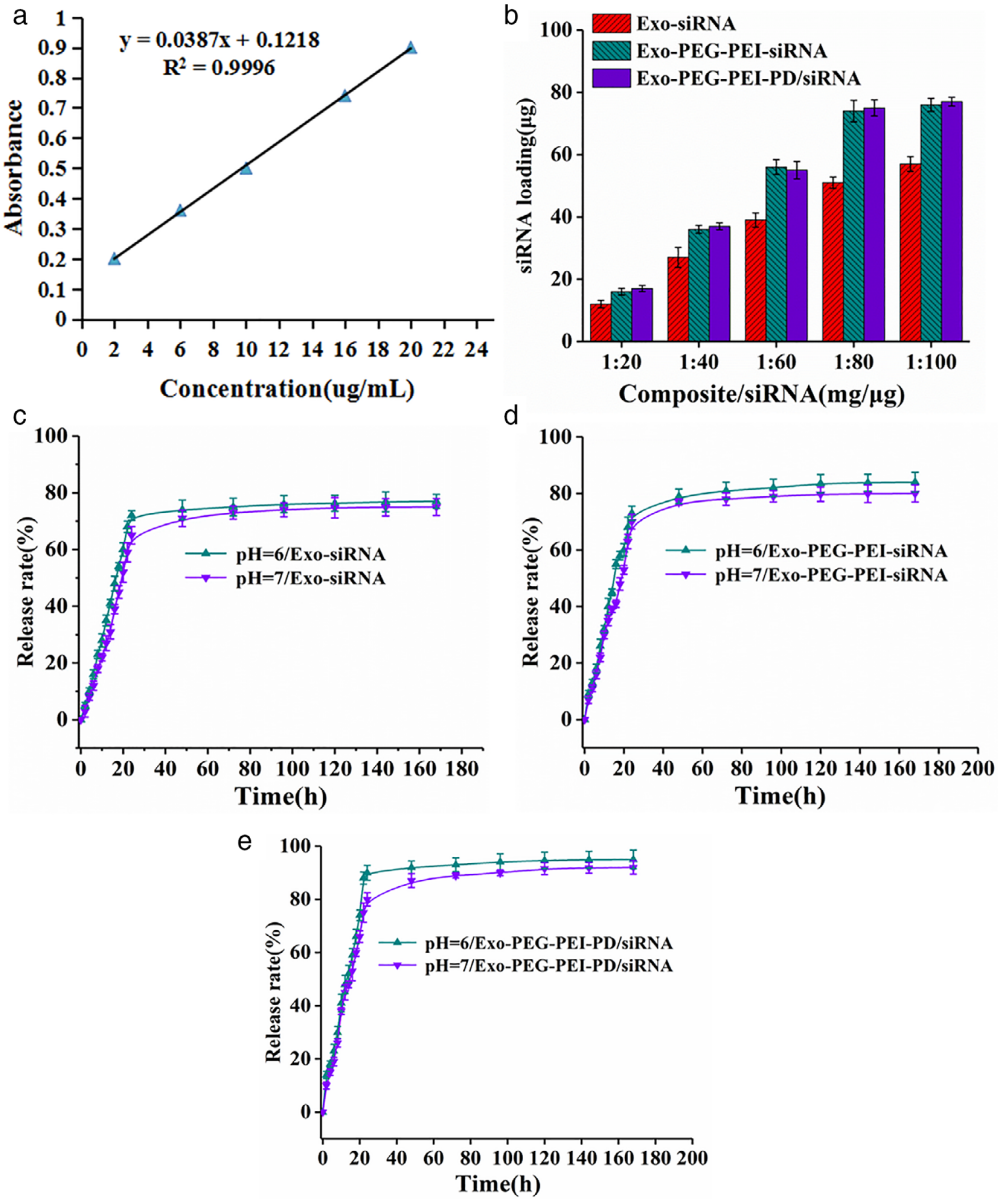
Loading, release, and cell uptake assay. (a) siRNA standard curve. (b) siRNA loading. (c) Release rate of Exo‐siRNA. (d) Release rate of Exo‐PEG‐PEI‐siRNA. (e) Release rate of Exo‐PEG‐PEI‐PD/siRNA

**FIGURE 3:**
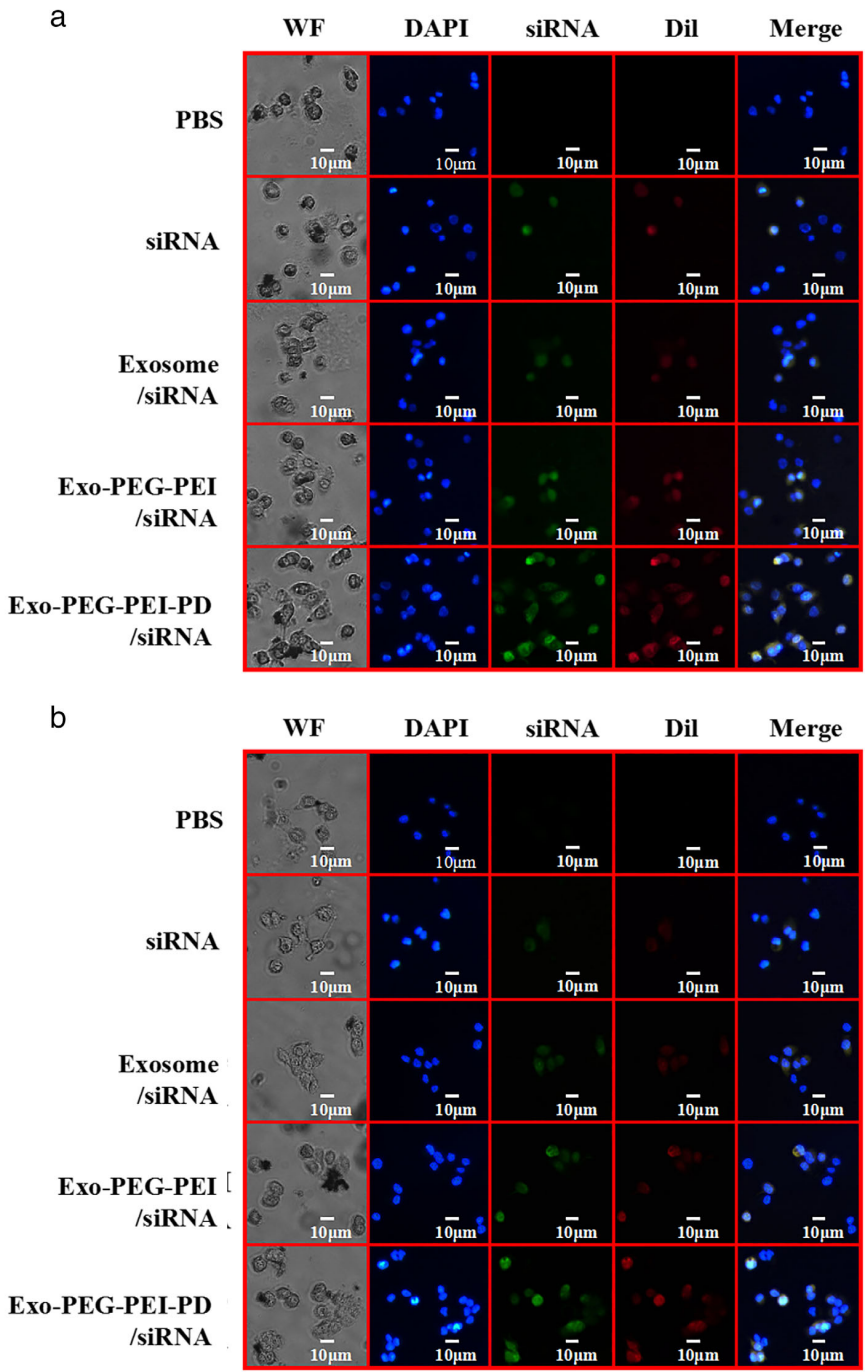
Cell uptake experiment. (a) A549 cell uptake experiment. (b) H460 cell uptake experiment

A siRNA standard curve was tested using a UV spectrophotometer (Figure 2a). siRNA was 1 mg: 80 μg, and the loading rate of siRNA in Exo‐PEG‐PEI‐PD/siRNA was 93.75% (Figure 3b). The release efficiency of siRNA in Exo‐siRNA, Exo‐PEG‐PEI‐siRNA, and Exo‐PEG‐PEI‐PD/siRNA is shown in Figures 2c–e, respectively. The release curve was steep within the first 24 h and gradually turned into a gentle curve afterwards. The release rate of the loading system under acidic conditions was higher than at a pH = 7, and the release rate could reach 91.8% under acidic conditions. The protonation reaction of free primary amine groups on PEI in an acidic environment may have weakened the positive electricity of the polymer nanospheres, and the electrostatic interaction between the nanospheres and siRNA was weakened accordingly improving the release rate of siRNA.^24^, ^25^ The results of tumor cell uptake experiments showed (Figure 3) that siRNA, Exo‐siRNA, Exo‐PEG‐PEI/siRNA, and Exo‐PEG‐PEI‐PD/siRNA could all be effectively taken up by tumor cells, while Exo‐PEG‐PEI‐PD and the uptake efficiency of siRNA was the highest.

### Detection of cytotoxicity and proliferation

**FIGURE 4:**
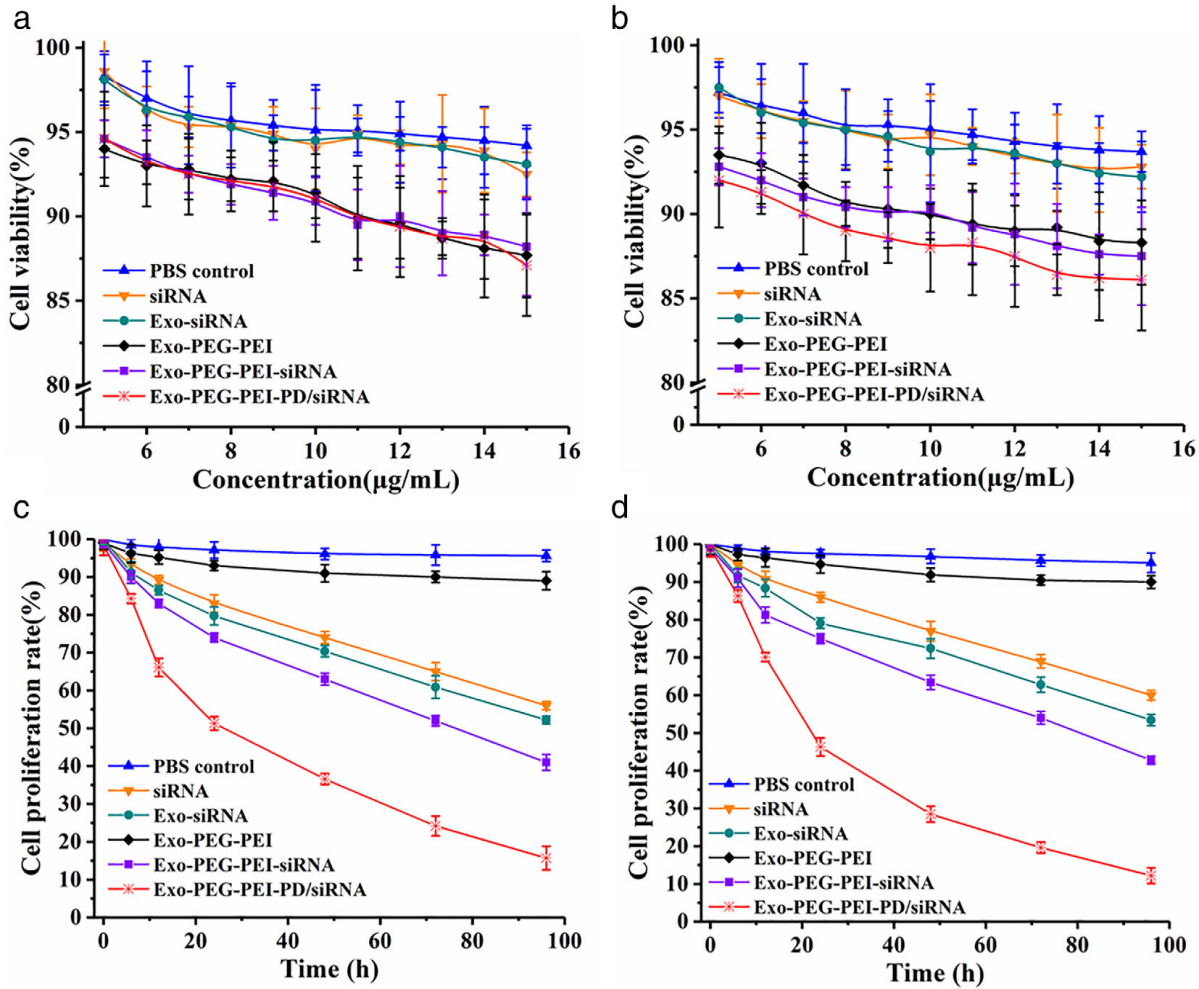
Detection of cytotoxicity and proliferation. (a) Detection of human umbilical vein endothelial cell (HUVEC) cytotoxicity. (b) Detection of A549 cytotoxicity. (c) Detection of A549 cell proliferation. (d) Detection of H460 cell proliferation

**FIGURE 5:**
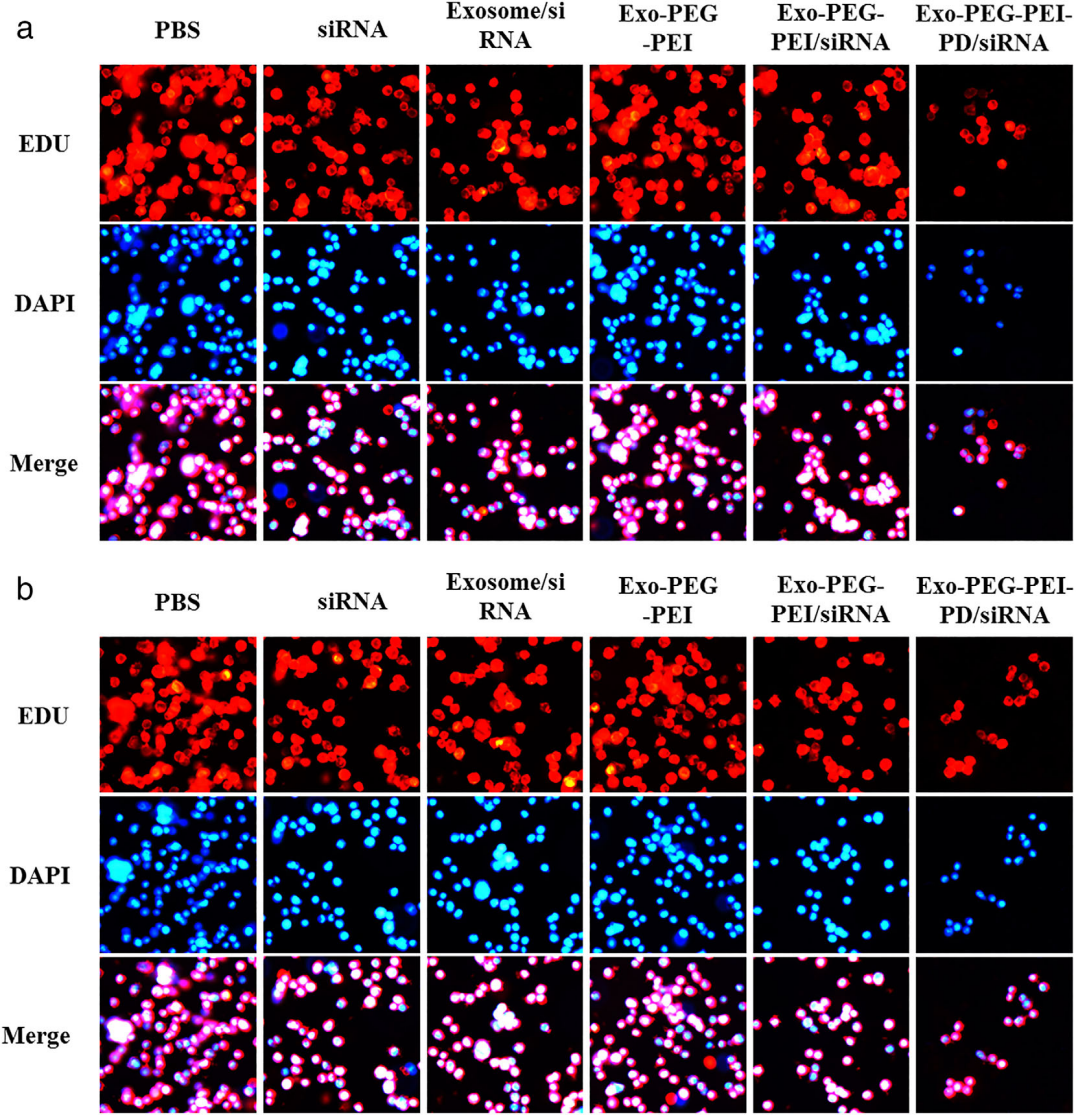
EdU method detects cell proliferation. (a) A549 cell proliferation detection. (b) H460 cell proliferation detection

SiRNA delivery is increasingly targeted at a single molecule level using biological coupling. Although such siRNA coupling showed potential for clinical research, positive carriers also demonstrate many problems like cytotoxicity of cationic materials, nonspecific adsorption of negatively charged cell membranes, and size inconsistency of nanoparticles and stability of nanoparticles in vivo. As a result, the in vivo application of DNA, RNA, and other biological agents have not been elucidated within the past 50–60 years.^26^, ^27^ The results of the experiment on the cytotoxicity of the materials prepared in this study showed that the cytotoxicity of Exo‐PEG‐PEI, Exo‐PEG‐PEI‐siRNA, and Exo‐PEG‐PEI‐PD/siRNA was low in HUVEC cells and that the cell survival rate was above 90% at a dosage <10 μg/ml (Figure 4a). In A549 cells, Exo‐PEG‐PEI‐PD/siRNA function targets and recognizes tumor cells, resulting in high cytotoxicity. When the concentration was <10 μg/ml, the cell survival rate was above 87% (Figure 4b). Low cytotoxicity is advantageous for gene therapy. The results of the cell proliferation experiment showed that siRNA, Exo‐siRNA, Exo‐PEG‐PEI‐siRNA, and Exo‐PEG‐PEI‐PD/siRNA can inhibit the proliferation of A549 and H460 cells. Since Exo‐PEG‐PEI‐PD/siRNA targeted identification, its inhibitory effect on tumor cell proliferation was more obvious. The proliferation inhibition rate of A549 and H460 cells reached ~50% 24 h later (Figures 4c,d). The results of EdU are shown in Figure 5a,b. Compared with the PBS group, the cell proliferation activity of the Exo‐PEG‐PEI‐PD/siRNA group was significantly reduced (*p* < 0.05). All of these experimental results showed that Exo‐PEG‐PEI‐PD/siRNA demonstrates an advantage of low cytotoxicity and functions in targeted identification and gene therapy. It can inhibit tumor cell proliferation and promote tumor cell apoptosis.

### Effects of apoptosis and cloning

**FIGURE 6:**
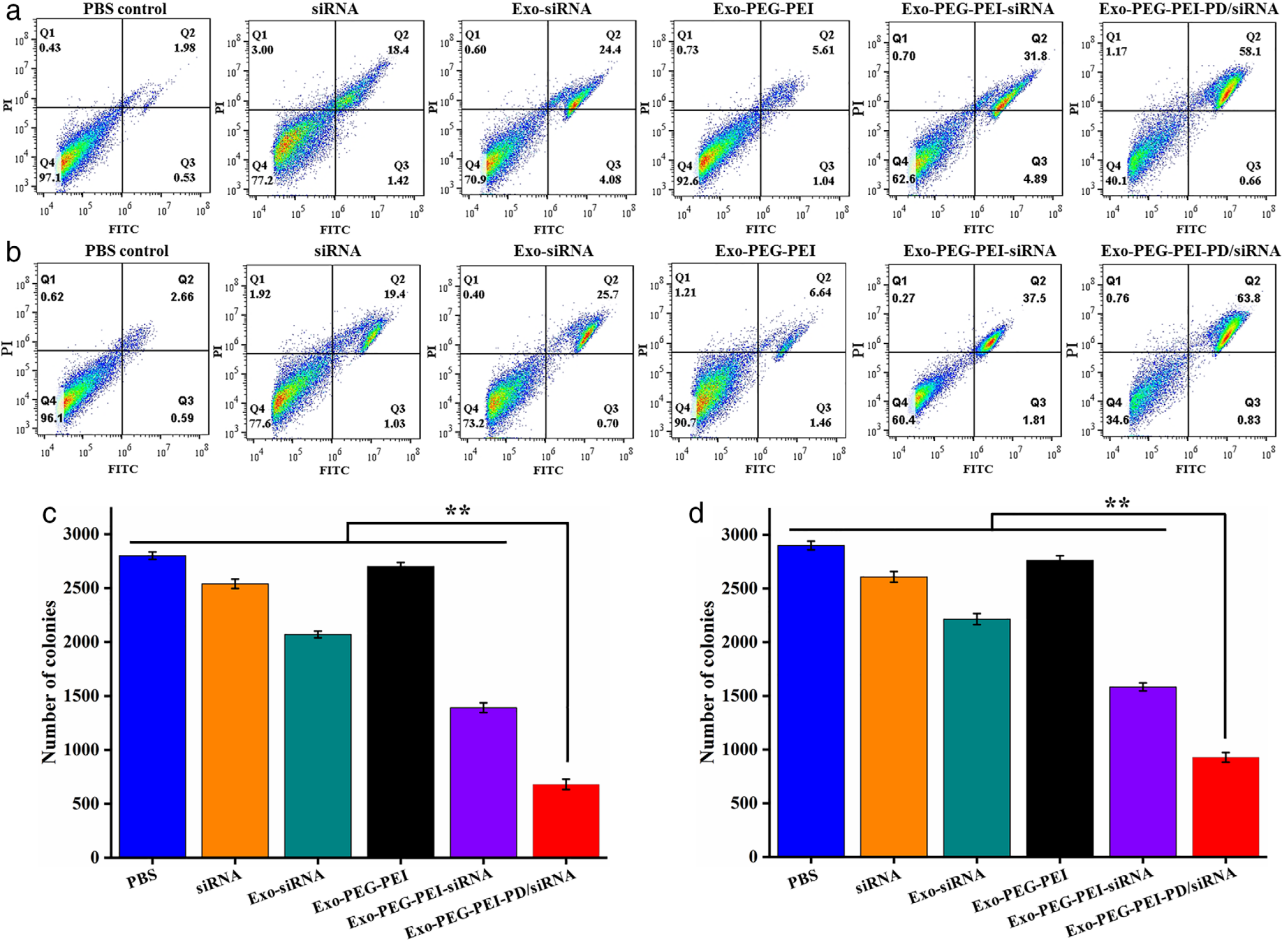
Effects of apoptosis and cloning. (a) Detection of A549 cell apoptosis. (b) Detection of H460 cell apoptosis. (c) Detection of A549 cell clones. (d) Detection of H460 cell clones

The results of apoptosis experiments showed that siRNA, Exo‐siRNA, Exo‐PEG‐PEI‐siRNA, and Exo‐PEG‐PEI‐PD/siRNA could all promote A549 and H460 cell apoptosis (Figure 6a,b). The cell cloning experiment results showed that siRNA, Exo‐siRNA, Exo‐PEG‐PEI‐siRNA, and Exo‐PEG‐PEI‐PD/siRNA could all reduce the cloning ability of A549 and H460 cells (Figures 6c,d). Compared to the PBS group, the cell cloning ability of the Exo‐PEG‐PEI‐PD/siRNA group was significantly lower (*p* < 0.05).

### PDL1 expression test results

**FIGURE 7:**
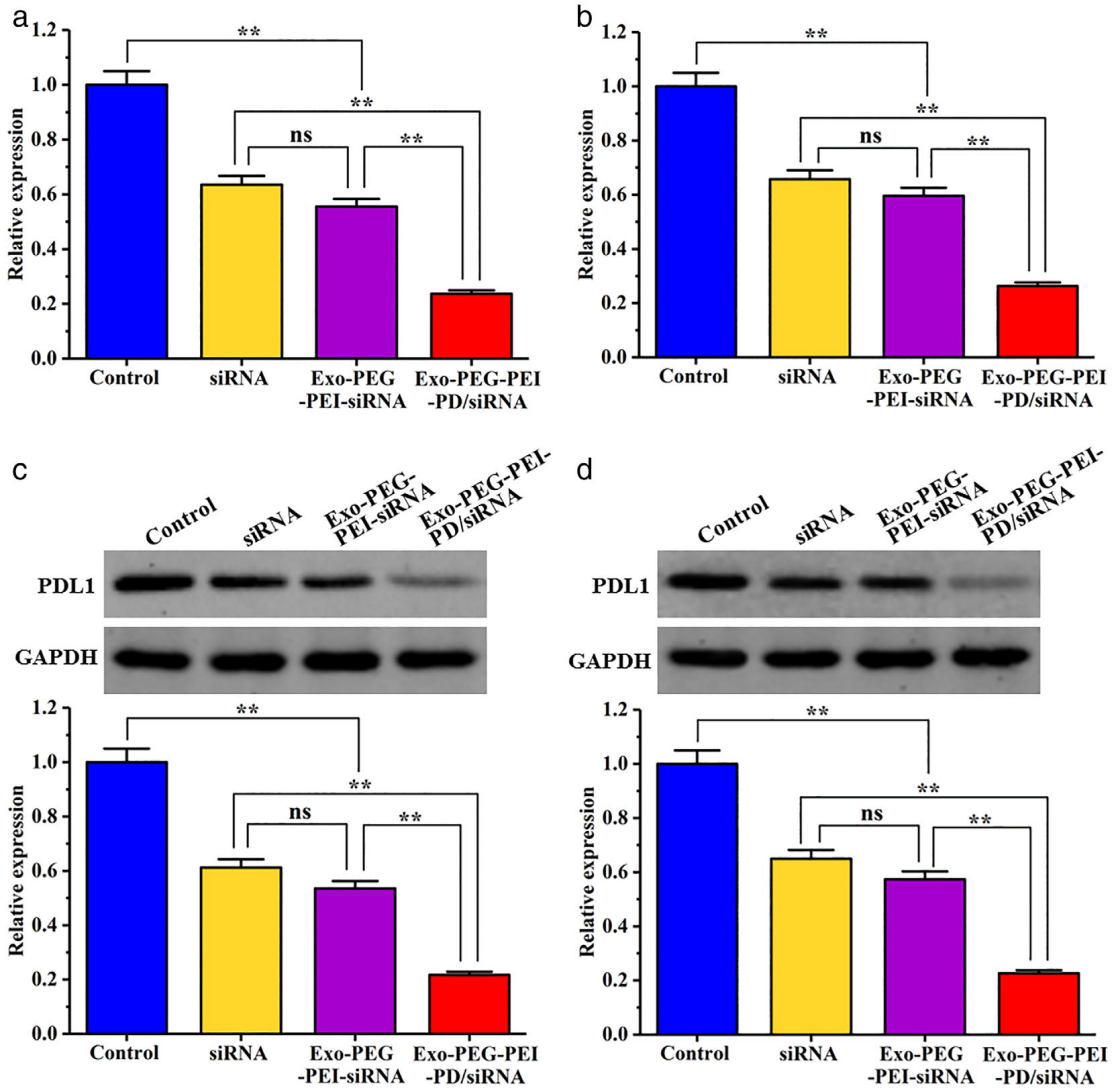
PD‐L1 expression in A549 and H460 cells. (a) PDL1 mRNA expression in A549 cells. (b) PDL1 mRNA expression in H460 cells. (c) PDL1 protein expression in A549 cells. (d) PDL1 protein expression in H460 cells

The results of the qPCR experiments are shown in Figure 7a,b. A549 and H460 cells in the blank group highly expressed PDL1 mRNA. The PDL1 mRNA expression was downregulated in cells treated with PDL1 siRNA, and the PDL1 mRNA expression in the cells treated with Exo‐PEG‐PEI‐PD/siRNA were significantly lower than the blank group. Western blot results are shown in Figure 7c,d. The blank group A549 and H460 cells highly expressed the PDL1 protein. In PDL1 siRNA treated cells, PDL1 protein expression was downregulated. For the Exo‐PEG‐PEI‐PD/siRNA group the expression of the PDL1 protein in treated cells was significantly lower than the blank group and the PDL1 siRNA group. It shows that PDL1 siRNA can inhibit the expression of PDL1, while loading siRNA through the PDL1 targeting complex can enhance the downregulation of PD‐L1.

## DISCUSSION

The use of extracellular vesicles as a vehicle for biological therapy is a promising strategy. Exosomes are extracellular vesicles that have been extensively studied. They carry proteins and nucleic acids and demonstrate many regulatory effects on recipient cells. They exhibit the ability to penetrate the interstitium of organs and natural targeting ability, and this makes them particularly attractive for the modification of pharmaceutical proteins and nucleic acids (such as siRNA). In particular, due to their endogenous origin, compared with exogenous nanobubbles, exosomes evade immune recognition and clearance.^28^, ^29^ As the endogenous carrier of the body, exosomes have the advantages of high biocompatibility, low immunogenicity, and inherent cell targeting, which can make up for the disadvantages of liposomes, siRNA conjugates, siRNA aptamers and polymers, and are expected to become a novel carrier for siRNA delivery.^30^, ^31^, ^32^, ^33^, ^34^ In addition, exosomes can be secreted by different types of cells in vitro, including immune cells, tumor cells, stem cells, hematopoietic cells, neuronal cells, and fibroblasts, and are also widely distributed in various body fluids (Table 1).^35^, ^36^, ^37^, ^38^, ^39^, ^40^, ^41^, ^42^ Aqil et al.^43^ demonstrated that exosomes can deliver endogenous RNA loads to recipient cells. siRNA‐loaded with exosomes is stable and resistant to degradation, and in various cancers, the expression level of siRNA against target genes decreased by 2–10 times. This current study utilized exosomes as carrier materials and modified them with PEG‐PEI to prepare exosome‐loaded exogenous siRNA targeting complexes with a PDL1 targeting function. Through the detection of physical parameters such as particle size and potential, it showed that the complex particles were small and relatively stable, with long circulation, a positive charge, and could be loaded with siRNA for gene delivery. The PD‐L1 antibody was successfully coupled to make the exosomes demonstrate higher tumor targeting and recognition capabilities. The optimal preparation process and binding ratio of the targeted nanovesicle/siRNA complex was determined through in vitro experiments. The release rate of the loading system under acidic conditions was faster than that at a pH = 7, and the release rate could reach 91.8% under acidic conditions.

**TABLE 1:** Application of exosome delivery of siRNA

Source of exosomes	Research content
Neuroblastoma cells	Treatment of human neuroblastoma with exosome‐tagged Hsp27 siRNA reduced the differentiation rate of mature neurons^32^
Autologous breast cancer cells	Exosome‐mediated siRNA delivery inhibits breast cancer postoperative metastasis^33^
Mesenchymal stem cell	Exosome‐modified tissue engineered blood vessels for endothelial progenitor cell capture and targeted siRNA delivery^34^
HEK293T	Engineering targeted tLyp‐1 exosomes as gene therapy vectors to efficiently deliver siRNA into lung cancer cells^35^
Epithelial MCF‐10A	In vitro and in vivo evaluation of functional exosome‐mimicking siRNA delivery to cancer^36^
Serum	Exosome‐mediated siRNA delivery for the treatment of inflammatory lung responses^37^
Plasma	Plasma exosomes deliver exogenous siRNA to monocytes and lymphocytes^38^
Cerebrospinal fluid	Exosome‐mediated delivery of hydrophobic modified siRNA for huntingtin mRNA silencing^39^

siRNA interference regulates the expression of RNA, selectively attenuates specific genes, and inhibits the function of specific RNAs. It has become a valuable research tool in cell biology and is also considered a possible treatment for many diseases. Since siRNA or shRNA is targeted, the use of siRNA or shRNA to inhibit specific RNA is a powerful cancer treatment strategy. Zhao et al.^44^ developed biomimetic nanoparticles (CBSA) combined with siS100A4 and exosome membrane‐coated nanoparticles. In vivo studies showed that CBSA/siS100A4@Exosome demonstrates a high affinity for the lung and exhibits a significant gene silencing effect, which inhibits the growth of malignant breast cancer cells. Tao et al.^45^ used ultrasound to synthesize exosome‐coated bcl‐2 siRNA that can effectively penetrate cell membranes, inhibit the growth of cancer cells, lead to apoptosis, and significantly inhibit the migration and invasion of cancer cells by downregulating metastasis‐related genes. In vivo studies showed that it significantly inhibits tumor growth in nude mice. Targeting siRNA tlyp1, exosomes can knock out the target genes of cancer cells, reduce the stemness of cancer stem cells, and can be used for gene therapy with high transfection efficiency.^38^ Lin et al.^46^ used iRGD modified exosomes to specifically deliver siRNA to tumors to inhibit FAO, reverse oxaliplatin resistance, and inhibit tumor growth, and they are safe in vivo. In this study, the Exo‐PEG‐PEI‐PD/siRNA complexes were subjected to a series of in vitro experiments such as cytotoxicity, tumor cell recognition, and inhibition to evaluate the function of the constructed complex drugs. Experimental results showed that siRNA, Exo‐siRNA, Exo‐PEG‐PEI‐siRNA, Exo‐PEG‐PEI‐PD/siRNA were less toxic to HUVEC cells, and the cell survival rate was above 90% when the dosage is 10 μg/ml. This inhibited the proliferation and cloning of A549 and H460 cells and promoted cell apoptosis. However, Exo‐PEG‐PEI‐PD/siRNA can target and recognize tumor cells due to the PD‐L1 antibody coupled on the surface, and it is effectively taken up by tumor cells. The inhibitory effect on tumor cell toxicity, proliferation, and clonality is more obvious, and it can effectively promote apoptosis of tumor cells.

In conclusion, exogenous siRNA can be loaded and delivered into target cells using targeted exosomes. Selective gene silencing can inhibit tumor cell proliferation and promote tumor cell apoptosis. This method exhibits potential for tumor immunotherapy and gene therapy. A new PGE‐PEI‐based gene delivery system is proposed to solve the challenges faced by gene carriers in vivo and demonstrates the physical and chemical properties required for in vivo gene delivery. It can effectively inhibit lung cancer cells and is an efficient and safe targeted therapeutic nanocarrier, indicating that exosomes can be used as efficient delivery carriers for siRNA. siRNA delivery using exosomes may be an effective treatment strategy against cancer.

## CONFLICT OF INTEREST

The authors do not have any competing interests to declare.
